# First detection of equine hepacivirus RNA in *Stomoxys calcitrans* (Diptera, Muscidae) in eastern Austria

**DOI:** 10.1186/s12917-025-04890-x

**Published:** 2025-07-17

**Authors:** Vicky Frisch, Anna Sophie Ramsauer, Irina Preining, Maria S. Unterköfler, Hans-Peter Fuehrer, Martin Hofer, Manolis Lyrakis, Emilie Bouhsira, Emmanuel Liénard, Jessika-M. V. Cavalleri

**Affiliations:** 1 Equine Internal Medicine, Clinical Centre for Equine Health and Research, Clinical Department for Small Animals and Horses, University of Veterinary Medicine Vienna, Veterinärplatz 1, Vienna, 1210 Austria; 2 Institute of Virology, Vetsuisse Faculty, University of Zurich, Winterthurerstr. 266a, Zurich, 8057 Switzerland; 3 Parasitology, Centre of Pathobiology, Department Biological Sciences and Pathobiology, University of Veterinary Medicine Vienna, Veterinärplatz 1, Vienna, 1210 Austria; 4 Shared Facilities, VetCore, University of Veterinary Medicine Vienna, Veterinärplatz 1, Vienna, 1210 Austria; 5 Platform for Bioinformatics and Biostatistics, Department of Biological Sciences and Pathobiology, University of Veterinary Medicine Vienna, Veterinärplatz 1, Vienna, 1210 Austria; 6 InTheres, Université de Toulouse, INRAE, ENVT, Toulouse, 31076 France

**Keywords:** Hepatitis, Horse, *Stomoxys calcitrans*, Stable fly, Mechanical vector, Muscomorpha, Pool feeder, Transmission, Equine, Hepacivirus, Flaviviridae

## Abstract

**Background:**

Equine hepacivirus (EqHV) from the *Flaviviridae* family, has been detected in horses worldwide with a global RNA prevalence of up to 7.9%. While vertical transmission and iatrogenic transmission with infected blood products have been demonstrated for this virus, field infection rates suggest an additional horizontal transmission route. The aim of this study was to investigate the potential role of *Stomoxys calcitrans* (Diptera, Muscidae) – a hematophagous fly that is found in stables with ruminants and horses as preferred hosts– in the transmission of EqHV RNA.

**Materials and methods:**

From 2021 to 2022, *S. calcitrans* were collected from three horse barns in eastern Austria. The abdomen of each fly was separated from the head and thorax. The heads and thoraxes, including wings and legs were subsequently pooled, with a maximum of five flies per pool, and assayed for the presence of EqHV using a one-step RT-qPCR. For all positive pools, the corresponding abdomens were analysed individually using the same EqHV one-step RT-qPCR.

**Results:**

A total of 783 *S. calcitrans* were collected at the three locations. EqHV RNA was detected in 7/136 pools of heads and thoraxes, including wings and legs, in 2021 and in 7/53 pools in 2022. Most positive pools were detected in autumn. The Ct values of the RT-qPCR were close to the presumed limit of detection. Additionally, EqHV RNA could be detected in 34 of 40 abdomens from 2021 to 20 of 40 abdomens from 2022, validating the results of the positively tested head/thorax pools. The minimum infection rate (MIR) was 1.2% in 2021 and 3.9% in 2022. The maximum likelihood estimation (MLE) was 1.2% in 2021 and 3.9% in 2022.

**Conclusion:**

Although the amounts of viral RNA were close to the limit of detection, the positive abdomens confirmed an up-take of virus-contaminated blood by the flies, and viral RNA residues were detected in the head and thorax. These results indicate that *S. calcitrans* may harbor EqHV in their head and thorax regions.

## Background

Equine hepacivirus (EqHV), also known as nonprimate hepacivirus or hepacivirus A, is a hepatotropic virus belonging to the family *Flaviviridae* which infects horses [1–3]. EqHV infections can result in hepatobiliary disease and may present as subclinical, acute or chronic persistent infections, frequently accompanied by slightly elevated liver- and bile-specific enzyme levels [3–8]. Although most described infections were subclinical and asymptomatic chronic infections leading to severe hepatitis with hepatic fibrosis and necrosis may be fatal [3, 7, 9, 10]. The economic risk of EqHV infections, potentially transmitted through contaminated blood products, includes substantial costs related to veterinary treatment and the potential loss of valuable horses [3, 11, 12]. Hepatitis C virus (HCV), the closest genetic relative of EqHV, is a well-known hepatotropic virus in humans and exhibits a comparable etiopathogenesis [3]. Investigations of EqHV may further advance the equine animal model for studying the biology and pathology of hepaciviruses, including HCV, which is already employed in vaccination studies [11–13]. The overall global prevalence of EqHV infections is 7.9% with rates as high as 16.1% in Asia and as low as 3.6% in Europe [11, 14]. In eastern Austria, EqHV RNA prevalence of 4.2% and antibody prevalence of 45.9% were reported in 2017 [15].

The minimum infectious dose of EqHV required to induce viremia was determined to be 13 RNA copies in a study where horses were inoculated with EqHV positive plasma [16]. This relative low dose, combined with the observed infection rates, suggests that in addition to confirmed iatrogenic (e.g., via infected blood products) and vertical transmission routes, horizontal transmission mechanisms may also play a role [11, 16–19]. For HCV, documented routes of transmission include contaminated blood products, inadequately sterilized medical equipment, sexual contact, and vertical transmission [20, 21]. HCV can survive on blood-contaminated surfaces for up to 16 h under favourable conditions [22]. Investigations of various equine secretions have detected EqHV RNA in nasal swabs, although these findings were not pursued further [23]. In contrast to other mosquito-borne flaviviruses, such as West Nile virus, viral RNA could not be detected in mosquitoes in Austria [15, 24, 25]. Studies on HCV have demonstrated its presence—and even in vitro replication—in certain mosquito species [26–29]. The transmission of HCV was detected in naive human blood after the transmembrane feeding of infected *Culex pipiens* s.l. [29]. These studies demonstrate the presence of viral nucleic acid and evidence of viral replication in mosquito cells but have not yet confirmed HCV vector transmission. These observations raise the possibility that the mechanical transmission via insect vectors could be an option for HCV transmission, which would be an even more relevant route for EqHV transmission in equids. Although the potential role of arthropod vectors, such as ticks and biting flies (including horse flies and stable flies), in mechanical transmission has been proposed this aspect remains insufficiently investigated and warrants further studies [15, 30, 31].

Several insect species can act as mechanical vectors and transmit pathogens via contamination or regurgitation of blood, secretions or excretions without supporting pathogen replication [32]. *Stomoxys calcitrans* (L.), (Diptera, Muscidae), is a known mechanical vector for several pathogens including the equine infectious anaemia virus [33, 34]. In contrast to most members of the Ceratopogonidae both male and female of the blood feeding Muscidae require bloodmeals, and *S. calcitrans* prefer to feed on livestock, equids or - if not available - other mammals and humans [35]. As pool feeders, their piercing-sucking proboscis creates a small wound in the host’s skin, enabling them to feed on the resulting blood pool [34, 35]. They are described as frequent feeders and feeding are often interrupted by their host, which forces the flies to search for a new host to complete the approximately 11 mg bloodmeal [32, 36]. Thus, contamination of the mouthparts and regurgitation of blood, increase the potential for pathogen transmission. Experiments using radiolabelled sucrose and blood have demonstrated that residues on mouthparts, along with the regurgitation of approximately 10% of ingested radioactive food, may facilitate the mechanical transmission of pathogens [37, 38].

The aim of this study was to screen the head and thorax regions including the wings and legs of *S. calcitrans* for EqHV to gain insights into its potential role as a mechanical vector for the virus.

## Results

### Sample collection / collection of flies

In 2021 and 2022, 783 *S. calcitrans* were caught in CO_2_-baited BG sentinel traps (Biogents, Germany) at three locations in eastern Austria, i.e. the University of Veterinary Medicine in Vienna (Vetmeduni campus) and two nearby villages located within a 30-kilometer radius from July to October (Table 1) [39]. In both years, at each location, two traps were set up for 24 h every 14 days. However, in October 2021, no traps were placed at two of the three locations, and in August 2022, an additional collection day was added. Equine clinical records from the University of Veterinary Medicine reported no confirmed cases of EqHV infection in horses from the study area during 2021 and 2022.

**Table 1:** Stomoxys calcitrans abundance as determined with BG-sentinel traps and EqHV infection rate in Austria in 2021/2022

	2021			2022		
	No of collections	No of flies collected	No of positive pools / No of pools	No of collections	No of flies collected	No of positive pools / No of pools
July: Total	**6**	**35**	0/8	**6**	**18**	1/6
Location 1	2	32	0/7	2	16	1/4
Vetmeduni campus	2	3	0/1	2	0	n.a.
Location 2	2	0	n.a.	2	2	0/2
August: Total	**6**	**84**	0/23	**7**	**15**	0/8
Location 1	2	5	0/4	2	12	0/3
Vetmeduni campus	2	10	0/3	2	2	0/2
Location 2	2	69	0/16	3	1	0/3
September: Total	**6**	**483**	7/105	**5**	**13**	3/8
Location 1	2	218	0/48	2	4	1/4
Vetmeduni campus	2	0	n.a.	1	7	2/3
Location 2	2	265	7/57	2	2	0/1
October: Total	**2**	**0**	n.a.	**6**	**135**	3/31
Location 1	0	0	n.a.	2	133	2/29
Vetmeduni campus	2	0	n.a.	2	2	1/2
Location 2	0	0	n.a.	2	0	n.a.
**Total no tested**		**602**	7/136		**181**	7/53

n.a. (no flies available)

Pool size varied, with each pool containing between one and five flies

**Fig. 1:**
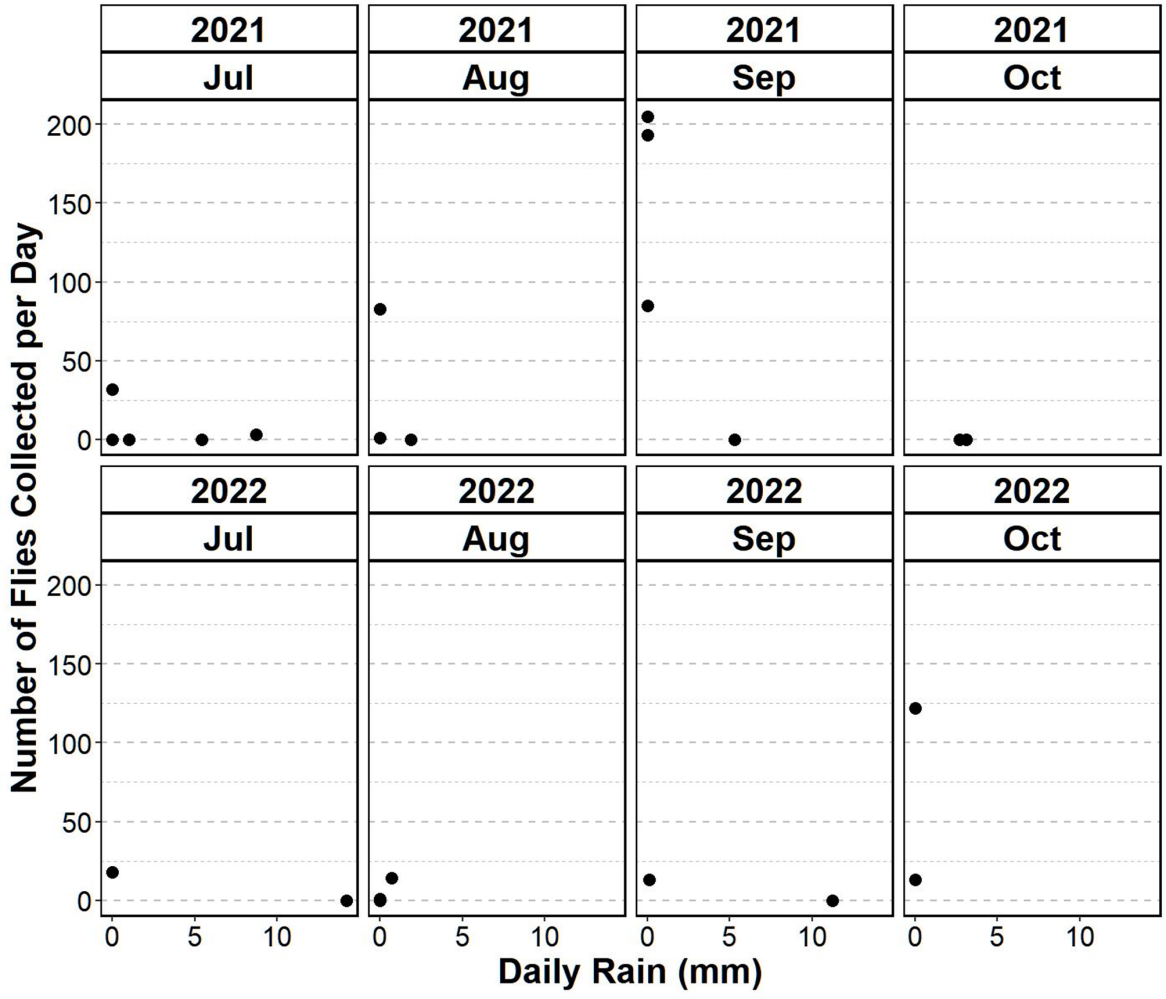
Comparison of daily rainfall (mm/day) and total number of *S. calcitrans* per collection day in 2021/2022

Information regarding the weather conditions during 2021 and 2022 in the area around Vienna, Austria, were retrieved from Open-Meteo [40]. The data includes daily mean, maximum, and minimum temperatures at 2 m above ground, along with total of daily rain in millimetres. A comparison of weather conditions during the study period revealed that 2022 was characterized by generally mildly warmer months. In 2021, the mean daily temperatures ranged from 7.3 °C to 26.9 °C, with maximum daily temperatures reaching up to 34.8 °C and minimum daily temperatures dropping to 1.7 °C. In 2022 the mean temperatures ranged from 9.4 °C to 27.9 °C, with maximum temperatures peaking at 35.3 °C and minimum temperatures falling to 4.4 °C. When comparing precipitation between 2021 and 2022, less rainfall was recorded in 2022 than in 2021 (Fig. 1). July and August were wetter in 2021 compared to 2022. July experienced a decrease in the number of rainy days and total rainfall in 2022. Similarly, August saw a reduction in both rainy days and total rainfall in 2022 compared to 2021. Conversely, September showed an increase in rainy days and total rainfall from 2021 to 2022. October consistently remained the driest month in both years.

Out of a total of 783 flies collected, 602 flies were collected in 2021, and 181 flies in 2022. Most of the flies were caught in autumn; in 2021 most of the flies were collected in September (*n* = 483) and in 2022 in October (*n* = 135), while no flies were caught in October 2021 (Table 1). Female and male flies were pooled separately. In 2021 out of 136 pools, 64 pools consisted of female flies (*n* = 272) and 72 pools consisted of male flies (*n* = 330). In 2022 out of 53 pools, 26 pools consisted of male flies (*n* = 87) and 27 pools consisted of female flies (*n* = 94). During the separation of the HTLW and the abdomen, varying degrees of specimen decomposing were observed after each sampling period of 24 h. Some specimens appeared to be engorged with red intestinal content, suspected to be blood, whereas others were dry and desiccated. Nevertheless, all specimens were treated equally, and a maximum of five HTLW were pooled for analysis.

### EqHV RT-qPCR positive HTLW pools

EqHV RNA was detected in pools of HTLW from *S. calcitrans* in both 2021 and 2022 (Table 1). At least two traps were operated at each location around the stables. In 2021, the flies that tested positive were caught in two different traps on the same day in September at Location 2. In 2022, positive flies were found in two different traps at Location 2 and at Vetmeduni campus, but at different times. In 2021, positive pools were detected only in September, whereas in 2022, positive pools were detected in July, September, and October (Table 1). The highest number of positive pools detected in the autumn of each year correlated with the high fly abundance during that period. In 2021 seven out of 136 pools tested positive, and in 2022, seven out of 53 pools tested positive for EqHV RNA. No differences were observed between female and male pools, as both male (*n* = 2) and female flies (*n* = 5) pools collected in 2021, as well as male (*n* = 6) and female (*n* = 1) pools collected in 2022, tested positive by RT-qPCR. In 2021, six of the seven positive pools presented at least 2 out of 3 positive replicates, with Ct values ranging from 37 to 40.7. One pool was positive only in 1 out of 3 replicates with a Ct of 40.7. In the re-screen, all pools presented at least 2 out of 3 positive replicates with Ct values ranging from 36.8 to 39.2. For 2022, low amounts of viral RNA could be detected, and only 1 out of 3 replicates was positive for each pool with Ct values ranging from 38.3 to 42.3 (Table 2).

During RNA isolation and RT-qPCR runs an isolation control, consisting of the HTLW of five naïve laboratory flies spiked with the same amount of EqHV-positive serum as the positive control was added. EqHV RNA was detected in the isolation control in all replicates (3/3) in each run. An inhibitory effect, with a difference between the mean values of 1.2 to 3.8 Ct values, was observed compared to the positive control.

**Table 2:** The validation of EqHV positive HTLW pools of *S. calcitrans* by RT-qPCR of the abdomen

Location	Trap emptying date	Sample name	Sample Description	EqHV RNA in HTLW		EqHV RNA in A	
			Number of HTLW F female M male	Replicates	Mean Ct	Positive samples*	Mean Ct range
Location 2	23.09.2021	1	5 F	3/3, 2/3	37.6, 38.1	9/10	33.6-37.9
		2	5 F	2/3, 3/3	37.6, 39.0		
		3	5 M	3/3, 2/3	37.0, 37.6	9/10	34.6-37.3
		4	5 M	2/3, 3/3	38.1, 36.8		
		5	5 F	2/3, 3/3	40.7, 36.8	10/10	33.9-37.3
		6	5 F	2/3, 3/3	37.4, 39.1		
		7	5 F	1/3, 3/3	40.7, 38.1	6/10	32.8-39.7
		8	5 F	Neg.			
Vetmeduni campus	22.09.2022	9	3 M	1/3	38.3	5/6	36.8-40.2
		10	3 M	1/3	42.3		
	08.10.2022	11	1 M	1/3	39.5	2/2	37.3-38.1
		12	1 F	Neg.			
Location 1	20.07.2022	13	2 F	1/3	41.3	1/2	37.1
	22.09.2022	14	1 F	Neg.	39.4	2/2	37.8-37.9
		15	1 F	1/3			
	08.10.2022	16	3 M	1/3	39.4	10/28	37.5-42.1
		17	3 M	1/3	38.0		

HTLW (head with thorax, legs and wings); A (abdomen only); Ct (cycle threshold)

*Positive samples from 2021 were rescreened and exhibited similar results upon the second testing

### Validation of positive HTLW pools by RT-qPCR of the abdomen

To confirm that the EqHV positive pools were from flies that had fed on EqHV positive blood and thus confirm the RT-qPCR results of the positive HTLW pools, the corresponding abdomen samples were analysed. The abdomens were tested individually to minimize inhibition in the RT-qPCR. The abdomens were pooled together for storage based on the date they were collected. As a result, the analysis included abdomens from negatively tested HTLW pools. This was because it was not possible to attribute the abdomens to specific HTLW samples. EqHV RNA was detected in 54 of 80 abdomens tested. In each positively tested HTLW pool, EqHV RNA was detected in at least one—and in some cases all—corresponding abdomen samples. The Ct values ranging from 32.62 to 42.12 were somewhat lower than those of the pooled HTLW (Table 2).

### Detection rate estimation

**Fig. 2:**
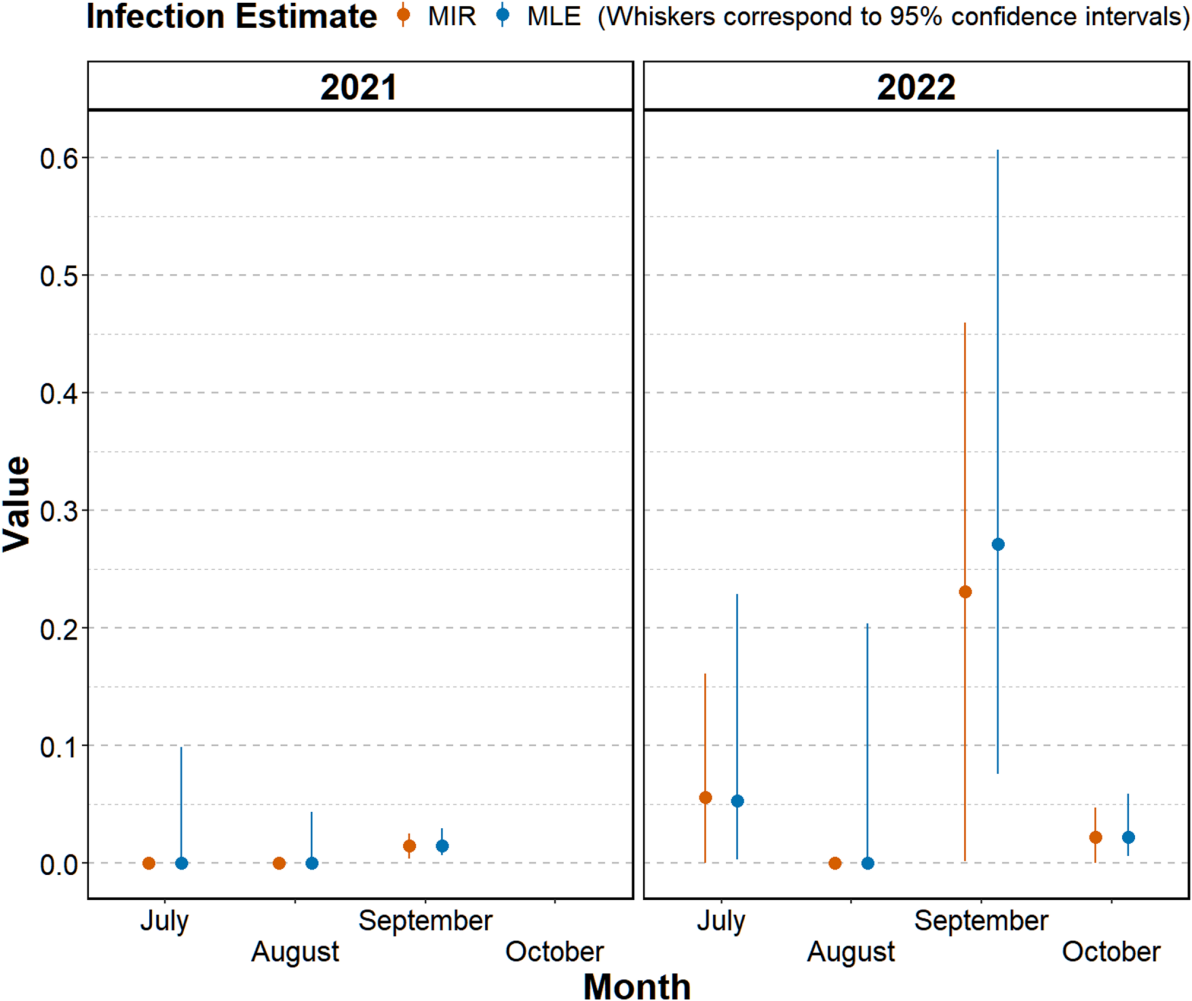
Monthly minimum infection rate and maximum likelihood estimation of EqHV in *S. calcitrans* in Austria during 2021/2022

To estimate the detection rate of EqHV RNA in the pooled HTLW samples of *S. calcitrans*, the minimum infection rate (MIR) and the maximum likelihood estimation (MLE) formulas were used [41, 42]. The MIR was calculated as 1.2% for 2021 and 3.9% for 2022. In 2021, the MLE was 1.2%, which was lower than the 3.9% reported in 2022 (contrast = -2.74%, 95% CI [-6.43%, -0.3%]). On a monthly basis, the highest MIRs and MLEs were obtained during September in both years (Fig. 2).

## Discussion

According to the equine clinical records at the University of Veterinary Medicine no cases of EqHV infections in horses have been reported in the study area during 2021 and 2022. Although the infection with EqHV is often asymptomatic, more severe forms of hepatopathies with fatal outcome are described [3, 7, 9]. To date, no specific therapeutic options or vaccines are available against EqHV. Investigating EqHV transmission routes is crucial for developing effective protective measures for horses. Only vertical and iatrogenic transmission routes have been demonstrated for this virus [11, 17–19]. Given the low viral copy numbers required for infection and the relatively high global antibody prevalences of up to 83.7%, another unidentified horizontal transmission route is suspected [9, 14, 43]. Given that EqHV belongs to the *Flaviviridae* family, we hypothesized that vector transmission, as with other members of the *Flaviviridae*, could be a possible transmission route [25]. To date, no EqHV RNA has been detected in mosquitos in eastern Austria [15]. We investigated hematophagous *S. calcitrans* as they are considered mechanical vectors for other equine viruses and are abundant in the region of eastern Austria. If *S. calcitrans* is confirmed as a mechanical vector, targeted interventions, such as reducing fly breeding sites to lower fly populations, could significantly reduce the risk of virus spread [35].

*Stomoxys calcitrans* has been implicated as a potential mechanical vector for various pathogens, including equine infectious anaemia virus [33]. The flies are pool feeders, cutting the skin with their piercing and sucking mouthpart, imbibing the emerging blood pool, and ingesting up to 15 µl of blood in 2–4 min [35, 36, 44]. The painful bite triggers defensive reactions in hosts, forcing the flies to find a new spot or even a new host, leading to a great exchange of flies between hosts [32]. After a bloodmeal, the flies can harbor blood residues on their mouthparts for up to 5 minutes [37]. The crop, located in the thorax, provides more favourable conditions for pathogen survival compared to the digestive tract and flies can regurgitate a significant amount of blood from the crop [32, 37]. Given the flies´ feeding behaviour, their potential role in mechanical EqHV transmission can be estimated by calculating viral load requirements and regurgitation volumes. In 2022 Gömer and colleagues showed that infected horses can reach viral titres of up to 10^8^ RNA copies per millilitre of blood, with only 13 RNA copies being sufficient to establish a high-titre infection [16]. Based on estimates that stable flies can regurgitate 0.1 µl during feeding, and assuming that this volume contains at least 13 RNA copies (equivalent to 1.3 × 10^5^ copies/ml), mechanical transmission would theoretically be possible given the high viremia in infected horses [16, 37, 38].

Higher numbers of flies 2021 were collected than in 2022, even though traps were set at only one of the three locations in October 2021, whereas in 2022, traps were set at all three locations. Furthermore, in August 2022, a third collection day was added at Location 2. Most of the positive tested HTLW pools were detected in autumn in both years. The seasonal pattern was anticipated based on the sampling data, as the highest number of flies were caught in September 2021 and October 2022 (Table 1). Similar observations were published from southwestern Slovakia, which is regionally close to eastern Austria [45]. *Stomoxys calcitrans* exhibited a flight season from May to October, with two peak periods: one in late summer and a second smaller peak in autumn [45]. A limitation of our study is that fly collection did not cover the entire active season. The lack of spring data is due to the fact that flies were obtained as bycatch from a mosquito study which only started in July of both years. Future studies should consider regional factors such as weather and seasonal variation affecting the flight activity of flies.

Comparison of weather conditions indicate that 2022 experienced generally mildly warmer months, and less rainfall than did 2021. All of these factors could influence the behaviour of the flies, affecting both the number of flies caught and the detection rates of EqHV RNA over the four months [35, 45]. Feeding behaviour and therefor flight activity of the flies is temperature-dependent [35]. During this study, temperatures dropped below 15 °C in September and October of both years, possibly reducing flight and biting activity [35]. Most of the pools were caught on days when the mean temperatures ranged between 9.2 °C and 23.3 °C. In October 2021, no flies were caught in the traps, which coincided with the lowest mean temperatures. In 2021, most of the flies were caught in August and September, and in 2022, most in October following a wetter period. Most of the pools were caught on days with low precipitation. Although precipitation can increase the potential ovipositioning sites for these flies it potentially hinder their flight activity [45–47].

EqHV RNA was detected in pooled HTLW samples and abdomens of *S. calcitrans*. Although EqHV RNA was detected in field-collected flies no conclusions regarding the presence of viable virus or transmission potential can be drawn. The methods used did not show any evidence of EqHV viral infection in *S. calcitrans*, which would indicate biological transmission. In this study the HTLW was separated from the abdomen to avoid mere xenomonitoring by detecting digested EqHV RNA positive blood in the flies´ abdomens. We wanted to investigate the contamination of the mouthparts, legs, and thorax, including the crop, an organ from which the flies can regurgitate contaminated blood [37, 38]. The detection of EqHV RNA in the abdomens, corresponding to the positive tested HTLW pools, verified our positive results. The positive tested HTLW pools prove contamination with viral EqHV RNA from this body regions and that not only contaminated blood was digested in the abdomens of the flies. To investigate a potential mechanical transmission route, studies are needed in which *S. calcitrans* transmits infective EqHV resulting in a viremic horse or using of artificial feeders.

Unlike mosquitoes, both male and female *S. calcitrans* consume blood meals, so no significant differences between female and male pools were expected [35]. Consistently, separate screening of the biological sexes revealed detectable levels of EqHV RNA in both. In contrast to our study, which analysed only *S. calcitrans*, EqHV RNA could not be detected in mosquitoes caught in the same region in Austria three years earlier [15]. In the broader context of various insect species and their potential role as virus vectors, no published evidence currently supports the transmission of EqHV via horse flies or other candidate vectors. HCV, the closest phylogenetic relative to EqHV, has been detected in mosquitoes collected from homes of HCV-positive individuals, and replication in various mosquito cell lines has been demonstrated [27–29, 48]. Neither mechanical nor biological transmission of HCV via mosquitos has been demonstrated. Although other blood sucking flies have been suggested as potential vectors for HCV, no studies confirming this have been published to date [30, 49]. Since *S. calcitrans* can use humans as hosts when preferred hosts such as livestock and horses are not available, a potential for cross-contamination merits further investigation [32, 35]. Horses can potentially serve as animal model to detect transmission routes that have not yet been explored [11, 13]. In addition to confirming the role of *S. calcitrans* in EqHV transmission, future studies should evaluate its potential as a mechanical vector for HCV and other pathogens.

No studies evaluating the survival time and infection capacity of EqHV outside its host have been published to date. HCV, has been shown to survive from 16 h to six weeks at room temperature [22, 50]. HCV was also shown to remain infective after inoculation with various body fluids such as saliva, tears and semen for up to 3 weeks [51]. If EqHV shows capacities similar to those of HCV and can remain infective in different body fluids such as tears over a prolonged period of time, other horizontal ways of transmission not just blood contamination should be considered. Furthermore, unlike HCV in humans—where transmission routes such as unsafe injection practices linked to drug consumption and sexual contact in high-risk groups are well documented—these routes are far less common in horses, except for iatrogenic transmission of EqHV [20, 21]. Although the high prevalence of EqHV in horses cannot be fully explained by the use of blood products, potential vectors may play a more significant role in EqHV transmission in horses than analogous insect vectors do in human HCV transmission. It is possible that *S. calcitrans* or another, yet unidentified, vector could help explain the observed transmission rates in the equine population. The evaluation should not only focus on *S. calcitrans* as a potential mechanical vector, but also on other insects such as *Musca autumnalis* (Diptera, Muscidae), commonly known as the face fly, whose females feed on the tear fluid of horses [34]. Thus, future investigations should examine whether tear fluid contains viral RNA, as this could offer insights into potential additional transmission routes.

Despite the lower fly numbers collected in 2022 the EqHV detection rate was higher. In 2022, positive pools were detected over three months (July, September, October), whereas in 2021, positive pools were detected only in September. Since no horses were tested during this study, we cannot make a statement on the active EqHV infections leading to RNA detection in flies. Since EqHV infection can persist in horses, the virus can overwinter in its equine reservoir. Thus, theoretically, when they feed on EqHV-infected horses, flies tested positive for viral RNA could be detected throughout the entire active fly season. No information about flies as potential reservoirs for EqHV could be retrieved from literature. It is assumed that flies can overwinter locally in stables and at different life stages due to the warmer climate and can continue to develop or reproduce in the spring [35, 52].

During our research, we demonstrated that the fly tissue inhibited the RT-qPCR assays. Previous studies have shown that whole flies, insect bodies and various mosquito body parts led to assay inhibition [53, 54]. We optimized the extraction and RT-qPCR protocol by including proteinase K, modifying the lysis protocol for the fly body and using a probe-based RT-qPCR-assay to increase specificity and sensitivity. After optimization, the control using spiked naïve laboratory flies still show an inhibitory effect, with mean value differences ranging from 1.2 to 3.8 Ct values. The flies were trapped in horse stables, resulting in a heterogeneous population of field collected *S. calcitrans* with varying body sizes and conditions. During homogenization, the bodies could not all be equally disrupted. This may explain the high Ct values detected in the samples and partial differences between pools. These results demonstrate that optimized sample processing is essential for achieving reliable outcomes and minimizing the risk of false negative results. Notably, the fact that viral RNA could be detected despite this inhibition may indicate an even higher viral load than initially assumed.

The RT-qPCR results approached the limit of detection, with cycle threshold values reaching as high as 43. The inhibition observed in the samples likely contributed to these high Ct values. This is consistent with the findings of Beckmann and colleagues (2012), who demonstrated that optimized analysis methods reduce inhibitory effects in mosquitoes, leading to an increased number of positive tested samples [53]. The HTLW of the flies were pooled into groups of five. Smaller pools may have favoured the outcome of our results. Another possibility is that the residues on the contaminated body parts of the flies, in the crop or abdomen, contained only a low amount of viral RNA. The viral load of the blood or body fluid residues, or the regurgitated previous meal, is the determining factor for mechanical transmission as no viral replication of EqHV is suspected in *S. calcitrans*. If the horses at the sampled locations exhibited low EqHV viral loads, the detection of low amounts of RNA in the flies is also expected. Another explanation is that the flies were not washed prior to lysis, leaving open the possibility of external EqHV contamination.

A limitation of our study is the calculation of a detection rate using the MIR and MLE, as we cannot prove an infection of *S. calcitrans* with EqHV. Although viral RNA was detected by RT-qPCR, it is not possible to cultivate this virus to date, and thus no conclusion can be drawn about an active infection. However, to better visualize the data and understand the EqHV RNA prevalence among the flies, despite pooling for analysis, the MIR and MLE were calculated. Given the small pools and the low RNA detection rates, the MIR and MLE do not differ significantly [41].

## Conclusion

This first detection of EqHV RNA in *S. calcitrans* may serve as an initial step in understanding transmission, while it provides limited evidence. Although the PCR results were consistently teetering the limit of detection-indicating very low viral loads-they do not prove that the flies are actively infected or directly establish a transmission route. Nonetheless, the detection of EqHV viral RNA in the head and thorax regions suggests that *S. calcitrans* may harbor EqHV. Further studies should focus on determining whether viable virus is present and whether these low viral loads are sufficient to cause infection, as well as to clarify the epidemiological relevance of such findings.

## Methods

### Collection and processing of flies

Both study periods started in July and ended in October for the years 2021 and 2022. During this time, two CO_2_ baited BG sentinel traps (Biogents, Germany) were set up for 24 h every 14 days at each location. An exception occurred in October 2021, when traps were not placed at two of the three locations, and in August 2022, when an additional collection day was added. The traps were operated according to the instructions provided in the user manual and were baited with CO_2_ released at 20.8 g / hour from a CO_2_ gas cylinder. The traps were set up in two horse stables (Location 1, Location 2) located within a 30-kilometer radius of the University of Veterinary Medicine Vienna, and at the Vetmeduni campus in Vienna [39]. Location 1 can accommodate a maximum of 62 horses, Location 2 eight horses and Vetmeduni campus approximately 100 equids. The equine clinical records at the University of Veterinary Medicine were reviewed for information about EqHV infections in horses presented at the equine clinic at the Vetmeduni campus between 2020 and 2023. The traps were installed for the purpose of monitoring West Nile Virus infections in mosquitoes. Along with mosquitoes, the by-catch collected in the traps was stored at -20 °C. The flies were collected dry, with no collection medium used in the traps to preserve their natural state. Based on morphological characteristics stable flies, *S. calcitrans*, were sorted from the pools and sexed. The abdomen of each fly was separated from the HTLW with a scalpel blade. A new scalpel blade was used for flies from each new date or location, and the blades were disinfected between every fly from one location and sample date. The body parts were stored separately by date in pools of five HTLW and pools of all abdomens from the respective date and trap. The body parts were stored at -80 °C until further processing.

### RNA isolation

To homogenize the flies, 180 µl ALT Buffer (QIAamp Viral RNA Kit, Qiagen, Germany) and two 3 mm Tungsten Carbide Beads (Qiagen, Germany) were added to the pooled HTLW as well as to the individual abdomen before lysing in the Qiagen TissueLyser II (Qiagen, Germany) for three minutes at maximum speed of 30 Hz (1800 oscillations/minute). After centrifugation, the supernatant was incubated with proteinase K for one hour at 56 °C followed by 10 min at 70 °C. After that, samples were processed using the QIAamp Viral RNA Kit (Qiagen, Germany) according to the manufacturer’s protocol.

### RT-qPCR

The NEB Luna Universal Probe 1-step RT-qPCR Kit (E3006E, New England Biolabs GmbH, Germany) combined with the equHepaci-Mix5-FAM targeting the NS3 region in the EqHV genome as described by Schlottau et al. 2019 was used [55]. A total volume of 15 µl was used for the reaction with 3 µl RNA template and 12 µl Master Mix composed of 7.5 µl Luna Universal Probe One-Step Mix (E3006E, New England Biolabs, Germany), 0.75 µl Luna Warmstart RT Enzyme Mix (E3006E, New England Biolabs, Germany), 0.9 µl of both forward primer 5136-F and 5128-F and 1.2 µl reverse primer 5212-R, 0.3 µl Probe Hepaci-5162-FAM [55]. The cycling template consisted of the reverse transcription for 10 min at 55 °C and inactivation for 2 min at 95 °C followed by 45 cycles of 10 s at 95 °C, 20 s at 56 °C, and 30 s at 60 °C performed on the qTOWER³ Real-Time PCR Thermal Cycler (Analytik Jena, Germany).

The positive control for the RT-qPCR consisted of EqHV RNA, which was isolated from 5 µl of virus-positive equine plasma (titer: 1.3 × 10⁵ RNA copies/mL, as reported by Gömer et al. 2022) using the QIAamp Viral RNA Kit (Qiagen, Germany) according to the manufacturer’s protocol [16]. Prior to the analysis of the flies from the three locations in and around Vienna, different RNA extraction protocols were applied to naïve strain of *S. calcitrans* from a laboratory colony established at the National Veterinary School of Toulouse, France, since 2009 (Team “resistance to insecticides”, JRU 1436 INTERES ENVT-INRAE) spiked with EqHV RNA isolated from EqHV positive equine serum to evaluate their efficacy. An inhibitory effect of the flies’ bodies on the isolation and RT-qPCR could be detected (unpublished data). To better evaluate the expected inhibitory effect of the flies´ bodies, a supplementary control was added to the first six viral RNA isolations and RT-qPCR runs. This consisted of HTLW of five naïve strain of *S. calcitrans*, from the previous used laboratory colony, processed in the same manner as the experimental samples, spiked with 5 µl of the same positive equine plasma as used for the positive control.

All the samples were evaluated in triplicate and the mean Ct was calculated to compare the results. Tests were considered positive if at least one of three replicates was below a Ct of 42.3. We considered values below 42.3 positive as this was the highest value achieved with a robust amplification curve. However, results near this value should be interpreted with caution and were therefore confirmed with the additional analysis of the individual corresponding abdomens.

### Statistical analysis

Statistical analysis was performed in R (R version 4.3.3) [56]. For visualization of the data and results we used ggplot2 (package ggplot2, Version 3.5.0, function ggplot) [57] and ggpubr (package ggpubr, Version 0.6.0) [58]. To estimate the detection rate of EqHV RNA in the pooled *S. calcitrans*, the MIR and MLE infection rates were calculated [41, 42]. The MIR and MLE infection rates and their 95% confidence intervals per month and year were estimated via the package PooledInfRate (Version 1.6, function pooledBin) [59]:

- General syntax: Number of positive pools ~ m(Pool size) + n(Number of pools)| Grouping variables separated with a star (*).
- Options for MIR: pt.method = “mir”, ci.method = “mir” (Wald binomial interval based on the MIR).
- Options for MLE: pt.method = “firth” (bias-corrected maximum likelihood estimate using Firth’s correction), ci.method = “skew-score” (skewness-corrected score interval) (default parameters).

Estimate contrasts between years and their 95% confidence intervals were also evaluated (package PooledInfRate, function pooledBinDiff, same syntax).

## Data Availability

The datasets used and/or analysed during the current study are available from the corresponding author on reasonable request.

## Abbreviations

ATLW: Abdomen with thorax legs and wings
Ct: Cycle threshold
EqHV: Equine hepacivirus
HCV: Hepatitis C virus
HTLW: Head with thorax, legs and wings
MIR: Minimum infection rate
MLE: Maximum likelihood estimation
RT-qPCR: Reverse transcription quantitative PCR
*S. calcitrans*: *Stomoxys calcitrans*
Vetmeduni Campus: University of Veterinary Medicine in Vienna
